# Simple method of saddle nose correction: A double-layer dermofat graft: case report

**DOI:** 10.1097/MD.0000000000030300

**Published:** 2022-09-02

**Authors:** Ho Yoon Jeong, Kyu-Sup Cho, Yong Chan Bae, Hyung Joon Seo

**Affiliations:** a Department of Plastic and Reconstructive Surgery and Biomedical Research Institute, Pusan National University Hospital, Busan City, Korea; b Department of Otorhinolaryngology and Biomedical Research Institute, Pusan National University School of Medicine, Pusan National University Hospital, Busan City, Korea.

**Keywords:** dermofat graft, nasal depression, saddle nose, saddle nose deformity

## Abstract

Saddle nose deformities are typically reconstructed with cartilage grafts; however, conchal cartilage grafts are and associated with a risk of damage to the posterior auricular ligament and insufficient amounts, and costal cartilage grafts require invasive surgery under general anesthesia. We proposed a double-layer dermofat graft as an alternative to these methods.

**Patient concerns:**

Two patients with type IV saddle nose deformity underwent reconstruction with nasal augmentation with a double-layer dermofat graft harvested from the gluteal sulcus.

**Diagnosis:**

After operation, photogrammetric analysis demonstrated an improvement in the dorsal depression area, which corresponded to the angle between the sellion, most depressed point, and pronasale. Rhinoplasty Outcome Evaluation questionnaire was assessed.

**Interventions:**

The graft was divided into 2 sections; the first section was implanted transversely into the depressed nasal framework, and the second section was inserted vertically from the nasion to the supratip break for augmentation.

**Outcomes:**

Both patients reported high satisfaction with the Rhinoplasty Outcome Evaluation questionnaire. The mean preoperative angle between the sellion, most depressed point, and pronasale was 157.8°, and the mean postoperative angle at 6 months was 176.9°.

**Conclusion:**

The simple method double-layer dermofat graft technique demonstrated excellent outcomes in saddle nose deformity correction, did not require cartilage, and was easily performed under local anesthesia.

## 1. Introduction

Saddle nose deformity is characterized by poor structural support of the nasal mid-vault. It may be caused by trauma, infection, or previous septorhinoplasty surgery.^[1–3]^ Correction of saddle nose deformity requires surgical intervention, but there is no consensus regarding treatment.^[4]^ Saddle nose deformities are often corrected with autologous grafts,^[1,5–9]^ such as septal, conchal, and costal cartilage and the calvarial and iliac bones, because these have the lowest infection and extrusion rates. Autologous tissue is easily harvested without critical donor site morbidity.

Different surgical methods are recommended depending on the severity of the deformity. Daniel and Brenner proposed that conchal and costal cartilage should be used for mild (type 2 and below) and severe (require reconstruction of the L-strut) saddle nose deformity, respectively^[10]^; however, it is difficult to acquire adequate cartilage from these sites. Harvesting conchal cartilage is also associated with a risk of injury to the posterior auricular ligament, whereas harvesting costal cartilage is invasive, requires general anesthesia, and associated with a risk for pneumothorax and chest wall deformity.^[1,10–12]^

Dermofat is an autologous tissue that is easily harvested in large amounts. Dermofat grafts are frequently utilized in rhinoplasty procedures, but few studies have been examined their role in saddle nose deformity correction, particularly in severe saddle nose deformity.^[4]^

This study presented a double-layer dermofat graft technique for saddle nose deformity reconstruction.

## 2. Patients and methods

### 2.1. Study design and population

This retrospective case series examined 2 patients who underwent surgical correction of type IV saddle nose deformity with a double-layer dermofat graft at the Pusan National University Hospital. The study was approved by the Institutional Review Board of Busan University Hospital and complied with the 1975 Declaration of Helsinki.

### 2.2. Operative technique

All procedures were performed by a single surgeon (H.J.S.). A skin and soft tissue envelope was elevated with a transcolumellar inverted V-shaped and bilateral marginal rim incisions. Dissection was continued in the supraperichondrial plane and extended in a cephalad direction. Through wide dissection, all structural deformities including deformed septum, upper lateral cartilages and lower lateral cartilages were exposed visually. The sizes and shape of depressed area were evaluated finally.

An elliptical dermofat graft measuring 7–8 cm × 1.5–2 cm was harvested from the gluteal region. The gluteal region was sufficiently thick, and the resulting scar was easily hidden under clothes. The skin was incised until the subcutaneous layer with a no. 15 blade, and the graft was de-epithelialized to prevent epidermal inclusion cyst formation.

**Figure 1.**
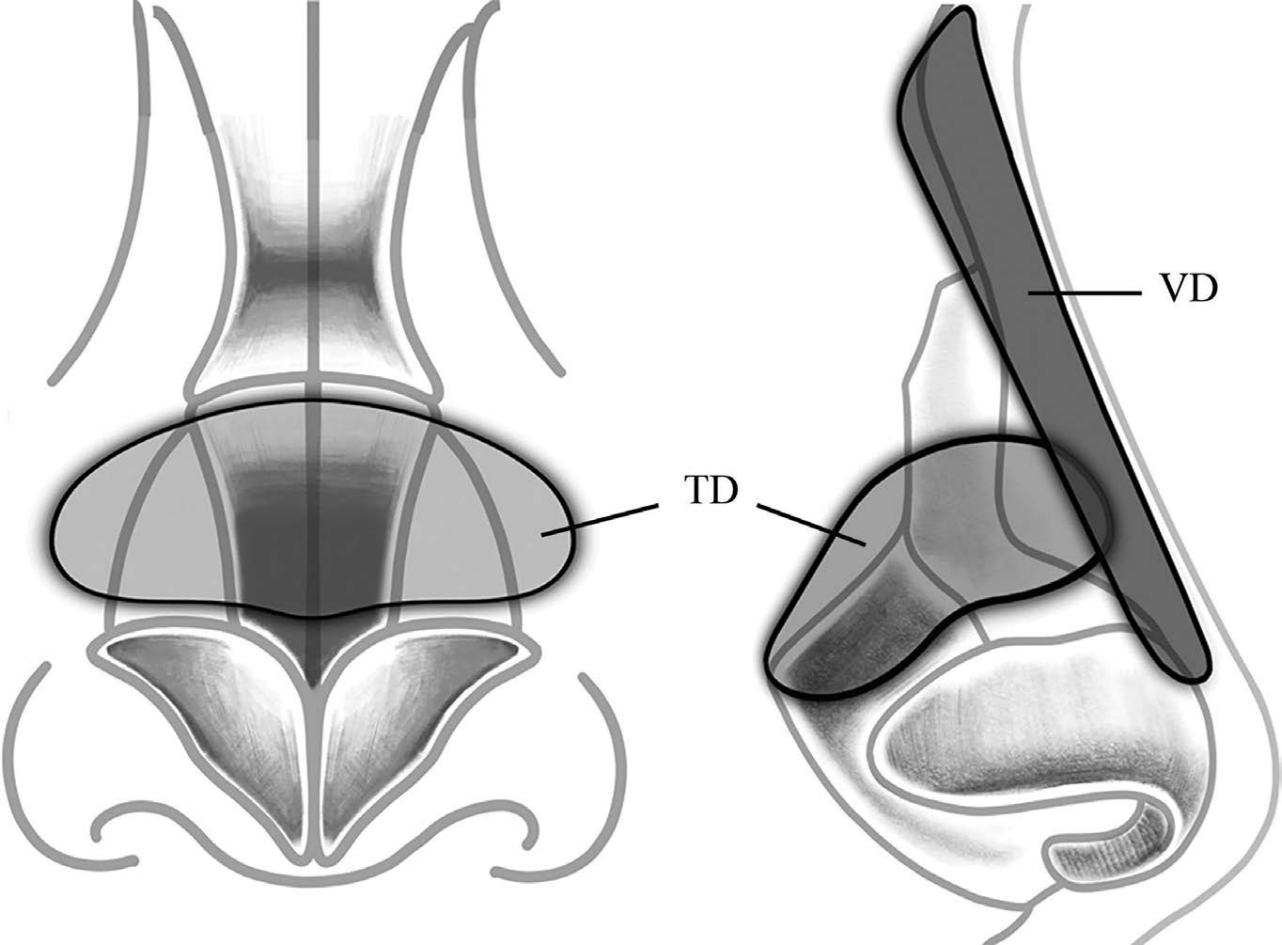
The dermofat graft harvested from the gluteal sulcus of the patient is divided into 2 sections. The transverse graft is transversely placed over the depression, and the vertical graft is used for additional correction of the depression and nasal augmentation from the nasion to the supratip. TD, transverse dermofat; VD, vertical dermofat.

The dermofat was divided into 2 parts, one is to correct the depressed area in transverse direction (transverse dermofat, TD), and the other is for the additional correction of depressed area with nasal augmentation in vertical direction (vertical dermofat, VD; Fig. 1).

The TD graft was shaped and inserted in the supraperichondrial layer of the depressed mid-vault and secured with 5-0 Ethilon (Ethicon, Cornelia, GA) sutures. The skin envelope was redraped to assess contour. The VD graft was sculpted to the desired dorsal width and height, similarly as in augmentation rhinoplasty. We targeted mild overcorrection to account for postoperative graft absorption. A 4-0 Nylon suture was placed at the cephalic end of dermofat and penetrated into nasal skin at the glabella midline through the 22G straight spinal needle. Next, the vertical dermofat was inserted into the prepared pocket with pulling out the suture. Finally, the other caudal side of dermofat was fixed with Ethilon 5-0 at the supratip area appropriately. The skin envelope was redraped to assess the final nasal shape and profile. The skin and mucosa were closes with simple interrupted sutures using 6-0 Ethilon (Ethicon) and 5-0 Vicryl. Merocele (Medtronic Xomed, Florida, USA) packing was performed in both nostrils, and a Joseph dressing with a thermoplastic external splint was applied for protection.

### 2.3. Assessment of surgical outcomes

**Figure 2.**
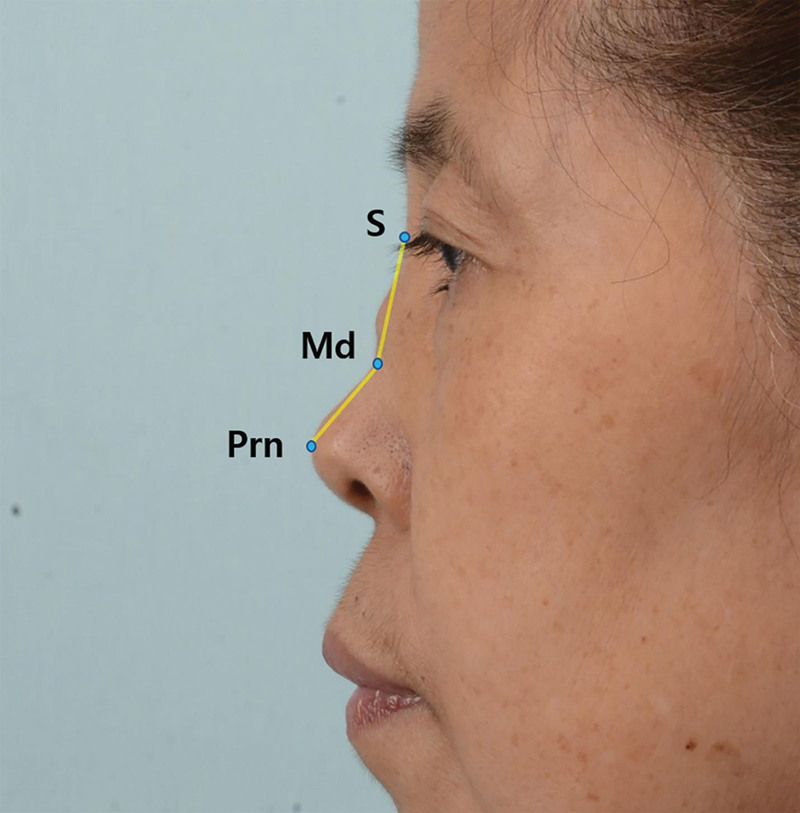
The dorsal depression angle is calculated with angular measurements and corresponds to the angle between the sellion, most depressed point, and pronasale on lateral view. S, sellion; Md, most depressed point; Prn, pronasale.

Surgical outcomes were assessed with preoperative and postoperative (at least 6 months) photographs, which were taken with a digital single-lens camera (Nikon d600; Nikon, Tokyo, Japan; focal length, 50 mm; aperture, f/9) with 2 flashlights. The dorsal depression angle corresponded to the angle between the sellion, most depressed point, and pronasale on lateral view (Fig. 2).

The Rhinoplasty Outcome Evaluation (ROE) questionnaire assessed patient preferences, perceptions, functional outcomes, and social appearance and was utilized to measure patient satisfaction. The ROE consisted of 6 categories. Each question was answered on a scale of 0 to 4, and the total score ranged from 0 to 24.^[13]^

### 2.4. Case 1

**Figure 3.**
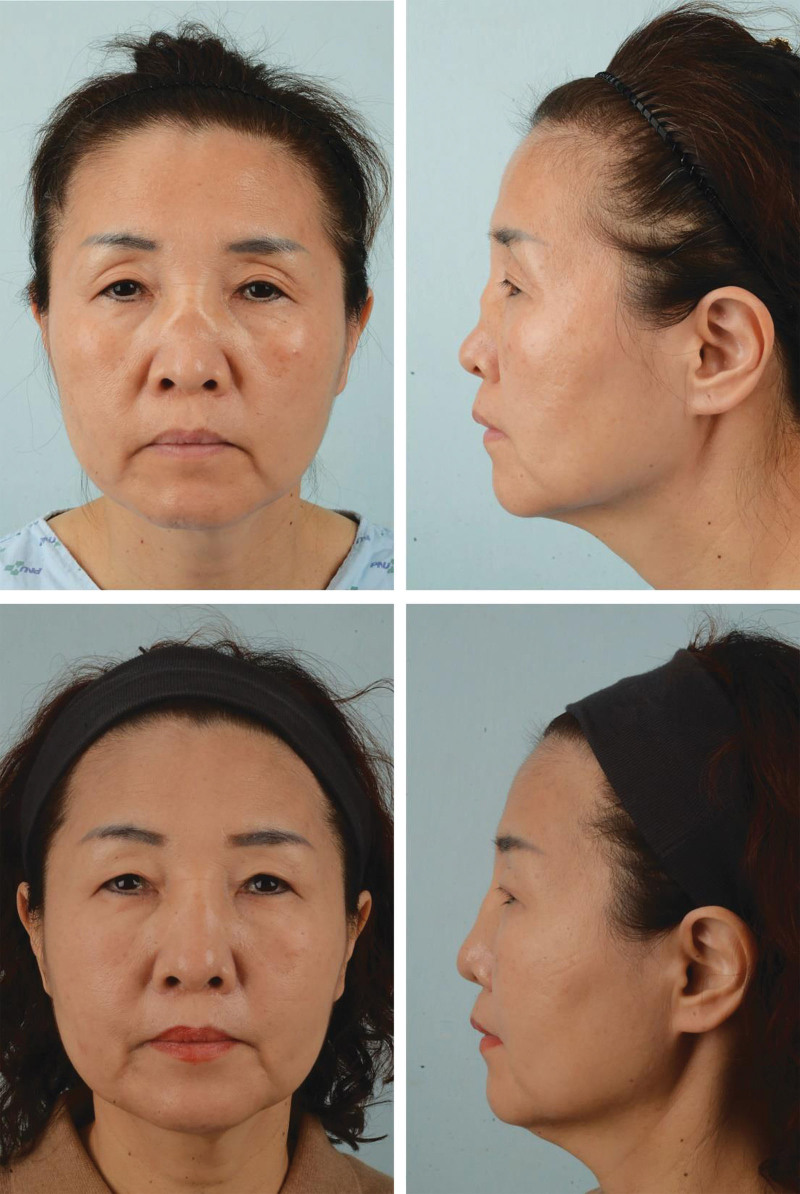
Preoperative (top) and postoperative 6-mo (bottom) frontal views. Preoperative mid-vault depression and inverted V-shaped deformity were corrected though double-layer dermofat grafting.

A 53-year-old woman with type IV saddle nose deformity had a preoperative dorsal depression angle of 159.6°. The patient declined reconstruction with costal cartilage but consented to saddle nose correction with dorsal augmentation with a double-layer dermofat graft. The operation was performed under general anesthesia due to endoscopic sinus surgery as a co-operation with the otorhinolaryngology department (Fig 3).

### 2.5. Case 2

**Figure 4.**
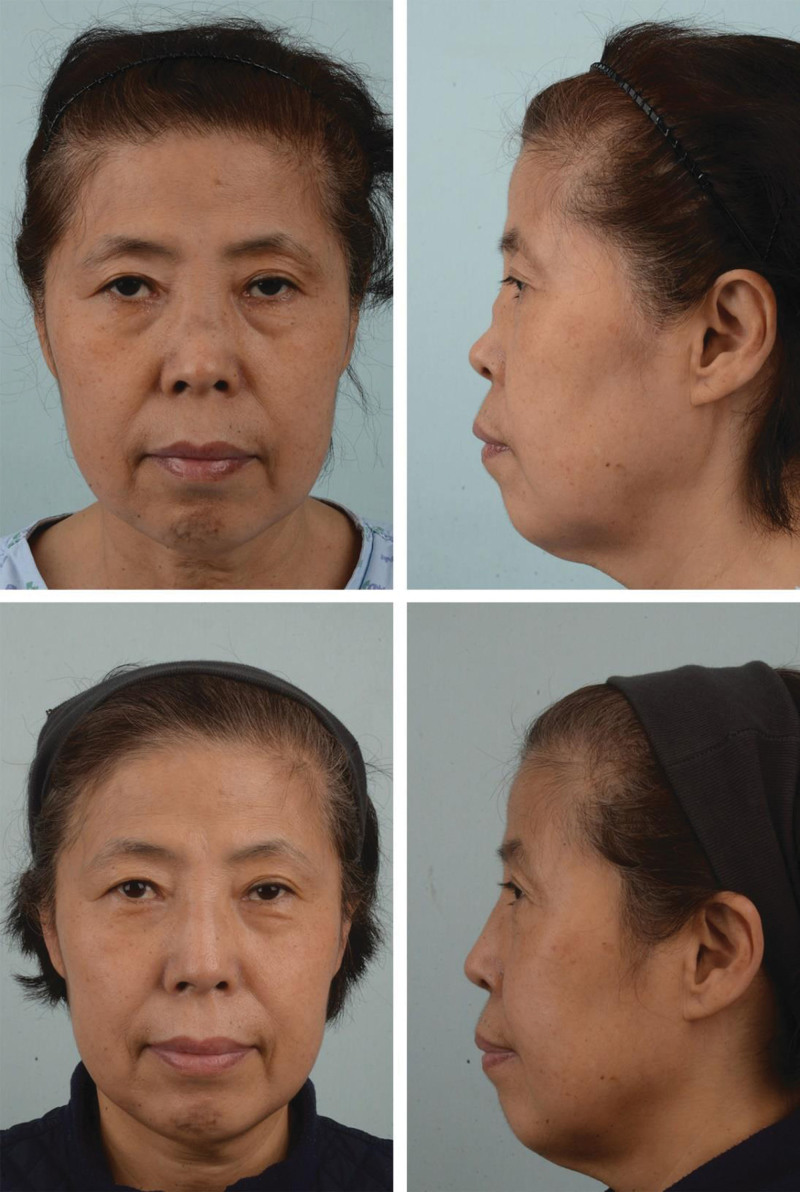
Preoperative (top) and postoperative 6-mo (bottom) profile views. The dorsal depression angle was 159.6° preoperatively and improved to 176.5° postoperatively.

A 59-year-old woman with type IV saddle nose deformity presented with endoscopic findings of chronic inflammation and large septal perforation from long-term steroid spray use and repeated intranasal procedures. Her preoperative dorsal depression angle was 156.0°. Saddle nose correction with nasal augmentation was performed under local anesthesia, because the patient had stage 5 chronic kidney disease and refused invasive surgery under general anesthesia (Fig. 4).

## 3. Results

No major complications, such as infection or hematoma, were observed during the 6-month follow-up period. Neither patient had cosmetic complications that required secondary surgery.

The preoperative dorsal depression angles of both patients measured 159.6° and 156.0°, and the postoperative dorsal depression angles of both patients at 6 months measured 176.5° and 177.3°. There was a significant improvement in the mean degree of dorsal depression from 157.8° preoperatively to 176.9° 6 months postoperatively. Both patients had ROE scores of 24, which indicated high satisfaction.

## 4. Discussion

The surgical procedure for saddle nose deformity correction is determined by the severity of the deformity and other patient factors.^[14,15]^ Grade 3 or higher severe saddle nose deformity requires L-strut reconstruction with costal cartilage;^[16,17]^ however, this requires an additional surgical site and general anesthesia. The double-layer dermofat graft technique is a promising alternative because it is less invasive and relatively simple.

Dermofat is an autologous tissue and has a relatively low risk for postoperative infection compared with artificial silicon blocks. Patients with saddle nose deformities commonly have a history of facial trauma or chronic inflammation, which increases the risk for thinning of the nasal skin or subclinical infection. Both of the patients in this study demonstrated chronic inflammation on endoscopy, but no complications, such as infection, were observed during the follow-up period.

The double-layer dermofat graft technique has several advantages. First, it provided excellent esthetic results because the TD graft addressed the depressed saddle deformity, and the VD graft provided simultaneous augmentation rhinoplasty. Performing the procedure with the TD graft alone would have resulted in a wide nasal vault, which led to patient satisfaction, while the VD graft effectively narrowed the dorsal width.

Second, the technique did not require conchal or costal cartilage. Grade 2 or lower mild saddle nose deformities are reconstructed with conchal cartilage,^[16]^ but the procedure often damages the posterior auricular ligament. The coronavirus disease 2019 era has made wearing masks indispensable, and insufficient structural support of the ear secondary to posterior auricular ligament injury worsens patient discomfort and causes limitations in daily life. Augmentation rhinoplasty is also difficult to perform with conchal cartilage because of inadequate tissue, whereas adequate dermofat can be harvested from a single donor site and utilized in dorsal augmentation and reinforcement of the thinned nasal skin.

Third, the technique was less invasive, required shorter operating times, and can be performed under local anesthesia. This technique is an option for patients who cannot be cleared for general anesthesia, such as in case 2. Performing the technique under local anesthesia also reduced financial costs.

Previous studies have shown that 15% to 45% of dermofat grafts are absorbed over time.^[18]^ Dermofat graft absorption may be reduced with the folding technique or overaugmentation. Kim et al proposed that minimal graft manipulation, meticulous tissue dissection with minimal reconstruction, and good bleeding control minimize dermofat absorption. We performed overaugmentation and meticulous dissection in both of the patients in this study, and neither required a repeat dorsal augmentation or revision rhinoplasty. If significant absorption was noted, a second-day fat graft was an option.

There were limitations in this study. Relative small sample size and insufficient follow-up period of <1 year should be improved though further studies. Despite these limitations, our study provides additional surgical option for the correction of saddle nose

In conclusion, the double-layer dermofat graft for saddle nose deformity correction was easily performed under local anesthesia. It did not require cartilage grafts, which risk injury to surrounding structures, and provided excellent esthetic outcomes and high patient satisfaction.

## Author contributions

Ho Yoon Jeong was responsible for data collection, data interpretation, manuscript writing, and approval of the final version. Kyu-Sup Cho was responsible for data collection and data interpretation. Yong Chan Bae was responsible for data collection and data interpretation. Hyung Joon Seo was responsible for study conception and design, manuscript critical revisions, and approval of the final version.
